# Determination of Specific Heat Capacity on Composite Shape-Stabilized Phase Change Materials and Asphalt Mixtures by Heat Exchange System

**DOI:** 10.3390/ma9050389

**Published:** 2016-05-19

**Authors:** Biao Ma, Xue-yan Zhou, Jiang Liu, Zhanping You, Kun Wei, Xiao-feng Huang

**Affiliations:** 1 Key Laboratory for Special Area Highway Engineering of Ministry of Education, Chang’an University, Xi’an 710064, Shaanxi, China; xueyan0229@163.com (X.-y.Z.); zyou@mtu.edu (Z.Y.); Weikun@chd.edu.cn (K.W.); xiaophenix@hotmail.com (X.-f.H.); 2 Architectural Design and Research Institute of Guangdong Province Xi’an Branch, Xi’an 710064, Shaanxi, China; liuj0817@163.com; 3 Department of Civil and Engineering Environment, Michigan Technological University, Houghton, MI 49931, USA

**Keywords:** road engineering, CPCM, specific heat capacity, heat exchange system, phase change material, asphalt mixture

## Abstract

Previous research has shown that composite shape-stabilized phase change material (CPCM) has a remarkable capacity for thermal storage and stabilization, and it can be directly applied to highway construction without leakage. However, recent studies on temperature changing behaviors of CPCM and asphalt mixture cannot intuitively reflect the thermoregulation mechanism and efficiency of CPCM on asphalt mixture. The objective of this paper is to determine the specific heat capacity of CPCM and asphalt mixtures mixed with CPCM using the heat exchange system and the data acquisition system. Studies have shown that the temperature-rise curve of 5 °C CPCM has an obvious temperature plateau, while an asphalt mixture mixed with 5 °C CPCM does not; with increasing temperature, the specific heat capacities of both 5 °C CPCM and asphalt mixture first increase and then decrease, while the variation rate of 5 °C CPCM is larger than that of the asphalt mixture, and the maximum specific heat capacity of 5 °C CPCM appears around the initial phase change temperature. It is concluded that the temperature intervals of 5 °C CPCM are −18 °C–7 °C, 7 °C–25 °C and 25 °C–44 °C, respectively, and that of the asphalt mixture are −18 °C~10 °C, −10 °C~5 °C and 5 °C~28 °C. A low dosage of 5 °C CPCM has little influence on the specific heat capacity of asphalt mixture. Finally, the functions of specific heat capacities and temperature for CPCM and asphalt mixture mixed with CPCM were recommended by the sectional regression method.

## 1. Introduction

Asphalt mixture is one of the more temperature sensitive mixtures, as high surface temperatures have a greater impact on the performance of asphalt pavement in regards to rutting, aging and fatigue [1]. Numerous investigations have found that the performance of asphalt can be highly affected by environmental factors, specifically temperature [2,3].

Phase change material (PCM) has the capability of storing and releasing thermal energy, so PCM can be used to store or release heat storage based on either the sensible heat principle or latent heat principle. Sensible heat is a specific thermal term that refers to a temperature rise due to heat absorption without the presence of a phase change. Latent heat refers to the absorbed or released heat during the process of a material’s phase transition under the condition of constant temperature [4,5,6]. Therefore, PCM can be used in an asphalt mixture to proactively regulate the temperature of asphalt pavement and improve the temperature performance of pavement. There have been numerous studies on incorporating PCM in asphalt mixture. Using the heat transfer theory and simulation test, Ma and Wang studied the effects of PCM’s thermo regulation on asphalt [7]. Numerous studies, carried out by Ma and Li, concluded that mixing organic solid–liquid PCM into an asphalt mixture was beneficial in adjusting the temperature of the asphalt pavement but it has negatively impacted the service performance of the asphalt mixture [8,9,10]. The addition of PCM to an asphalt mixture can change the working temperature and improve the temperature resistance capacity of asphalt mixture as well as its ability to adapt to changes in the environment [11]. Ma [12,13] prepared the composited shape-stabilized phase change material (CPCM), which was developed using Tetradecane, silica, Ethyl Cellulose (EC) and dispersant. Such CPCM adjusted the temperature of the pavement, increasing its adaptability to environmental changes. It can be applied directly to the asphalt mixture [14]. Therefore, CPCM was used as an admixture of asphalt mixture to improve the performance of asphalt pavement.

In previous studies [7,8,9,10,11,12,13,14], temperature changing behaviors of PCM were used to analyze the influence of CPCM on asphalt mixture. However, limited by the test conditions, a computer is needed to simulate the real working conditions of roads. Furthermore, the analysis on a simple temperature regulation of CPCM is not enough; the specific heat capacity is the critical parameter for thermal analysis. To indicate the influence of CPCM on asphalt mixture intuitively, studies of specific heat capacity on CPCM and asphalt mixture mixed with CPCM are in urgent need.

Endothermic and exothermic reactions are the essence of temperature change, so temperature can be expressed as a quantity of heat by calculating the specific heat capacity. There is no universal method for measuring specific heat capacity. Differential Scanning Calorimetric (DSC) [15] was used to test the phase change temperature, latent heat and thermal stability by measuring the physical appearance of the material with temperature change. Melting point, endothermic enthalpy, and exothermic enthalpy of docosane PCM were tested by DSC and Thermal Gravimetric Analysis (TGA). Alkan reviewed that the Microencapsulated phase change material using docosane as the core material has remarkable chemical stability and thermal stability [16]. Bo [17] studied liquid–solid phase equilibrium and phase transformation of Tetradecane and hexadecane binary mixtures by DSC. T-history was used to measure the solid point, specific heat, latent heat, thermal conductivity and thermal diffusion coefficient of phase change materials. The theory behind T-history testing of the latent heat of PCM is a comparison of the temperature history between the PCM and a reference material subjected to conditions capable of the lumped capacity method [18]. Zhang YP [19] studied the T-history method and stated that for the engineering and preparation of new PCMs, which is an especially useful method in choosing the best fit PCM.

In previous research, a Marshall specimen was used to determine the specific heat capacity of asphalt mixture by the solid standard test method [20,21]. After the temperature of water and heated containers stabilize, a low temperature object was put into hot water and the heat exchanged until the temperature equilibrated. The water temperature and the initial temperature of the low temperature object were known, and the specific heat capacity of solid was calculated using the heat balance law. In summary, DSC has advantages of high maturity and high precision. The advantages of the T-history method are that only a simple apparatus is required (sealed tubes) and no sampling requirements, which make it useful when measuring the thermo-physical properties of materials that exhibit inhomogeneous phase changes [22]. However, the integrity of the materials used in the T-history method was destroyed, likely to affect the measured data. As for solids with a quantity less than 20 mg and a particle size smaller than 6 mm, the most common method is DSC. As the quantity of solid ranges from 5 g to 10 g, T-history is more reliable.

The above test methods for specific heat capacity were just for a small quantity, which cannot be applied to test the specific heat capacity of solid materials with a greater quantity. As with the increasing of the sample quality, the peak shape of DSC curves moves to the right. The existence of a temperature gradient between the sample surface and the sample center, an increase in sample quality, an increase in the temperature gradient in the center of the sample center during the temperature-rise period, and an extended time of peak completed or transferred led to the overall peak shape shifting to the right [23]. In addition, asphalt mixture is mainly composed of aggregates of different sizes, which ranged from 0.075 mm to 13.2 mm in this study. When the aggregate size is large, the Marshall specimen must to be large enough to ensure uniformity in the specimen. DSC is unable to test large sized material, though T-history test can obtain the specific heat capacity of each composition of asphalt mixture, as there are many factors that affect the specific heat capacity of asphalt mixture so the specific heat capacity of asphalt mixture cannot be obtained by averaging the specific heat capacity of each material. Obviously, the two test methods cannot measure the specific heat capacity of asphalt mixture directly. Meanwhile, the solid standard test method was applied to the materials with relatively small temperature changes, and an accurate specific heat capacity was not obtained. The data were imprecise, the results were unstable, and the calculated data were scattered and unreliable. Above all, a new method is urgently needed to test the specific heat capacities of CPCM and asphalt mixture.

Therefore, this paper proposed a heat exchange system to test real-time temperature change behaviors and calculate the specific heat capacities of CPCM and asphalt mixture mixed with CPCM. The specific heat capacity was used to study the influence of CPCM on asphalt mixture.

## 2. Materials

**Figure 1:**
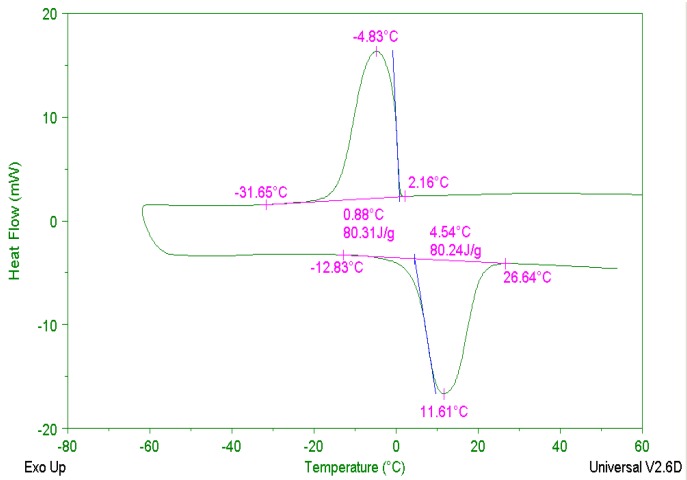
Differential Scanning Calorimetry (DSC) test results of 5 °C PCM.

The shape-stabilized PCM is made up of, in certain proportions, PCM and silica as a carrier material [12]. Ethyl Cellulose (EC) works as the membrane material and Ethyl alcohol (CH_3_CH_2_OH) as an organic solvent for EC to cover the PCM material. Plasticizer was used to improve the flexibility of the membrane material. The initial phase change temperature of CPCM used in this study was 5 °C, which is 5 °C CPCM for short. The DSC curves of 5 °C CPCM [14] are shown in Figure 1. During the exothermic process, the initial phase change temperature and final phase change temperature were 2 °C and −31 °C, respectively, and the compensation power peak appeared at −5 °C or so. The exothermic enthalpy was 80.31 J/g.

SBS (I-C) modified asphalt was selected as the original asphalt, which is asphalt modified by adding thermoplastic styrene-butadiene rubber (SBS) into the matrix asphalt while a percentage of the exclusive stabilizer is added to form SBS blend material. I-C indicates that SBS modified asphalt meet the I-C technical requirements of SBS modified asphalt; flash rock, mechanism sand and grounded limestone were selected as coarse aggregate, fine aggregate and slag, respectively. The technical indicators of the above materials were in accordance with the specification requirements [24].

## 3. Experiment Methods

The data were used to calculate the specific heat capacities of 5 °C CPCM and asphalt mixture mixed with 5 °C CPCM obtained from the heat exchange system and the data acquisition system. The details of the heat exchange test are as follows.

### 3.1. Heat Exchange Test

The heat exchange test was used to test temperature using a double insulation barrels system and real-time record temperature using a data acquisition system, which consisted of a Pt100 RTD sensor (32), XSL Series 32-channel data logging devices (Habiaorongda Automatic Measurement and Control Technology Co., Ltd., Beijing, China) and a computer.

#### 3.1.1. Principle and Determination of Specific Heat Capacity

The specific heat capacity is a thermal index, expressed by *c*, as seen in Equation (1).

(1) Q=cm∆T

In Equation (1), *Q* is the quantity of heat, *m* is the material mass and Δ*T* represents the temperature difference.

Based on the heat transfer theory, there will be a heat transfer phenomenon as long as a temperature difference exists. Heat continues to transfer until the temperature difference disappears. According to the energy conservation law, the heat released from a high temperature object is equal to the heat absorbed by a low temperature object, which is shown in Equation (2).

(2) $$c_{w}m_{w}∆T_{w}=c_{x}m_{x}∆T_{x}+Q^{\prime }$$

In Equation (2), *Q*’ is the heat loss during a heat exchange process, *c*_w_ is specific heat capacity of a high temperature object, the *c*_w_ under different temperatures are shown in Table 1. *m*_w_ is the mass of the high temperature object and Δ*T*_w_ is the temperature difference of a high temperature object. *c_x_* is the specific heat capacity of a low temperature object, *m_x_* is the mass of the low temperature object and Δ*T_x_* is the temperature difference of a low temperature object. In this paper, the high temperature object is water and the low temperature objects are the 5 °C CPCM specimen and the Marshall specimen. In this study, the initial specimen temperature is −20 °C and the initial water temperature is 60 °C.

The loss heat of system (*Q*’) in Equation (2) is characterized by a temperature diversification of the comparative barrel, which can be eliminated by a heat exchange temperature difference Δ*T*_E_. It is the temperature difference when water exchanges heat with 5 °C CPCM. As for the whole insulation system, the loss in water temperature during the heat exchange process is controllable. In each double insulation barrels system, the comparative barrel was used to amend the system heat loss. The temperature difference of the comparative barrel is heat loss caused by an endothermic system, while the temperature difference of the heat exchange barrel is caused by absorbing heat from hot water, which consists of system heat loss and the heating of PCM. Δ*T*_E_ is equal to the temperature difference of the heat exchange barrel subtracted from the comparative barrel, thus the actual heat loss can almost be eliminated and the water temperature change fully accounts for the sample temperature change. Therefore, the specific heat capacity can be calculated using Equation (3).

(3) $$c_{w}m_{w}∆T_{E}=c_{x}m_{x}∆T_{x}$$

#### 3.1.2. Heat Exchange Test Equipment 

**Figure 2:**
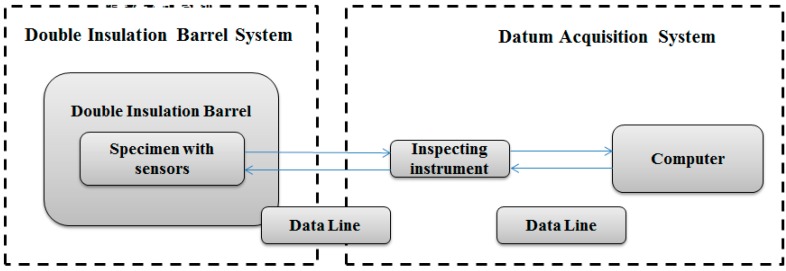
Heat exchange system.

The heat exchange test was simple to conduct, low in cost and well insulated. It was capable of measuring in detail the temperature change behaviors of the 5 °C CPCM specimen and the corresponding Marshall specimen. The heat exchange system is shown in Figure 2.

**Figure 3:**
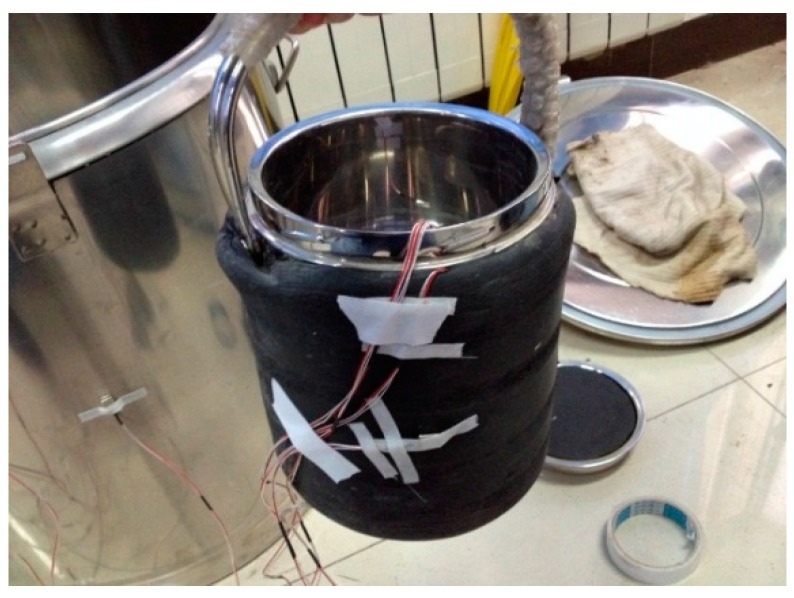
Little insulation barrel.

**Figure 4:**
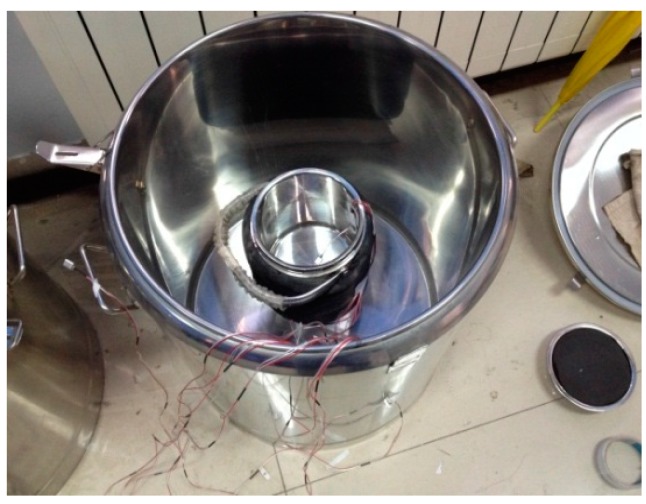
Double insulation barrels system.

The insulation barrel system consists of a little and a big insulation barrel. The little insulation barrel was used to exchange heat and was packed in 2 cm of cotton insulation, while the big insulation barrel was applied to stop the internal heat of the system from spilling out. The double insulation barrels system are shown in Figure 3 and Figure 4.

#### 3.1.3. Measuring Method of Real-Time Temperature

The determination process of real-time temperature is listed as follows.

**Figure 5:**
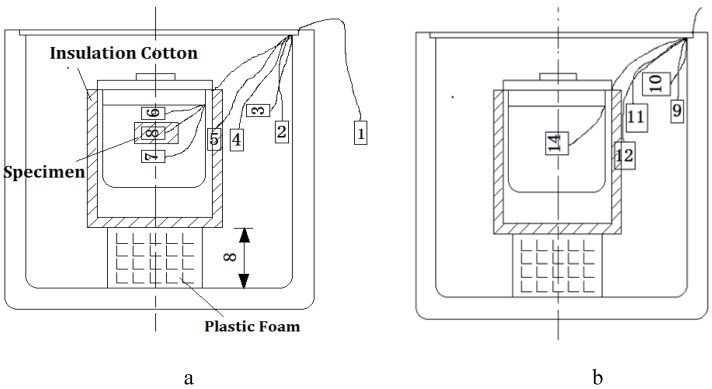
Diagram of the temperature sensor layout: (**a**) heat exchange barrel; and (**b**) comparative barrel.

(1) The heat exchange barrel was used to exchange heat from a high temperature object to a low temperature object; the comparative barrel was applied to correct the temperature loss caused by the system during the heating process.
  In Figure 5, sensor **1** was used to observe the temperature of the environment, sensor **2** and sensor **9** were used to observe the internal wall temperature of the two big insulation barrels, sensor **3** and sensor **10** were used to observe the cavity temperature of the two insulation barrel systems, sensor 6 and sensor **7** were used to observe the upper and lower temperature of the heat exchange barrel, sensor **14** was used to observe water temperature of the comparative barrel, and sensor **8** was used to observe the central temperature of the tested specimen.
(2) After placing the temperature sensors correctly, a certain quantity of hot water at 60 °C was poured into the two little insulation barrels, successively and quickly.
(3) Next, the specimen was placed into the little insulation barrel of the heat exchange barrel, then sealed and put into the big insulation barrel.
(4) The heat exchange process finished when the water temperature was equal to the specimen’s temperature.
(5) Finally, data were extracted from the computer to analyze the temperature change behaviors and calculate the specific heat capacity using Equation (3).

### 3.2. Water Temperature Correction

Even though the 5 °C CPCM increased by 1 °C, the temperature of the water in a comparative barrel and heat exchange barrel decreased by less than 0.1 °C. However, the precision of the temperature sensor used was 0.1 °C. Therefore, to improve the data precision, suitable functions were used to fit the change curves of the water temperature. The water temperature of the 5 °C CPCM specimen and asphalt mixture mixed with 5 °C CPCM are different from each other. Detailed functions displayed in Section 4.1.2 and Section 4.2.2, respectively.

### 3.3. Experiment Plan

The 5 °C CPCM specimen was made by a geotechnical mold with a 70 mm diameter and 35 mm height, then packed in storage bags and compacted in the geotechnical mold. The homogeneity of the 5 °C CPCM specimen is controlled by the volume of the geotechnical mold, and the 5 °C CPCM mass is 85 g. It is prepared at room temperature. There are five nearly identical samples when measuring the specific heat capacity of 5 °C CPCM. The water mass used to exchange heat is 1200 g.

The corresponding fraction of no-PCM samples are coarse aggregate, fine aggregate, artificial sand, mineral powder = 22%:34%:41%:3%. A 5 °C CPCM asphalt mixture was prepared by directly adding 5 °C CPCM to the asphalt mixture with a suitable temperature and dosage. The 5 °C CPCM was added by mass and the sample has the same mass by discarding excess after fabrication. The 5 °C CPCM asphalt mixture was designed based on the modified Marshall method [25]. Four different levels of mass concentration of the 5 °C CPCM in the asphalt mixture Marshall specimen were used to observe the temperature change behaviors and calculate the specific heat capacity of the asphalt mixture. The four detailed mass concentrations of 5 °C CPCM were 0.6%, 1.2%, 1.8% and 2.4%, respectively, represented by 1#, 2#, 3# and 4#. A temperature sensor was set in the middle of the Marshall specimen at a depth of 32–33 mm. The water mass used to exchange heat is 900 g.

## 4. Results and Analysis

### 4.1. Results and Analysis of 5 °C CPCM Specimen

#### 4.1.1. Temperature Changing Behaviors of 5 °C CPCM Specimen

**Figure 6:**
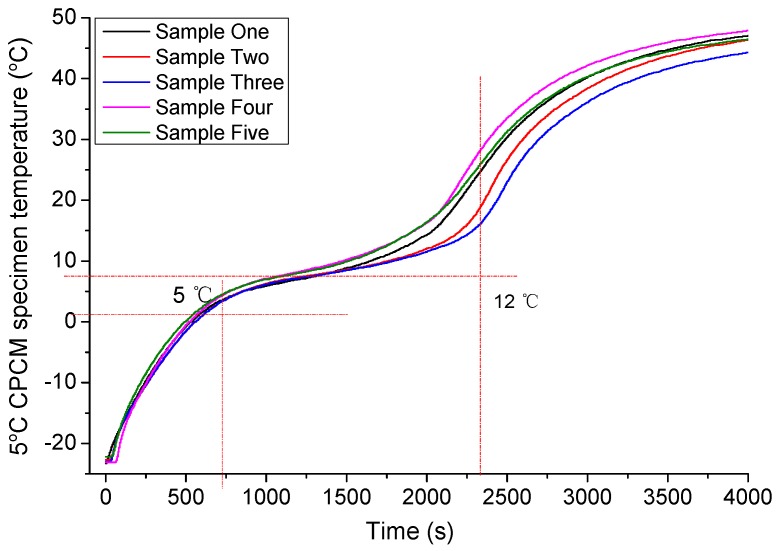
Temperature change behaviors curve of 5 °C CPCM.

From the heat exchange test, the temperature-rise curves of the 5 °C CPCM specimen are shown in Figure 6, and there are five nearly identical samples. In this study, the experiment was repeated five times with the five samples having about the same consistency.

The temperature change behaviors of the five nearly identical samples in the heat exchange process were similar. The curve consists of three arcs: the first and the third curves were both convex rising, while the second curve was concave rising. Based on the peak of the first arc and the second arc, the whole curve was divided into three sections, and the boundary temperatures were 5 and 12 °C, respectively.

In the first section, the temperature increased from its initial temperature to 5 °C. From the DSC curves of the 5 °C CPCM, the initial phase change temperature was about −10 °C. While in Figure 6, the phase change was slow and the material was an overall solid. The decrease in the rising rate is relevant to the reduction in the temperature difference between the water and the sample. During the second section, the temperature increased from 5 °C to 12 °C, the 5 °C CPCM temperature rose successively. From the DSC curves, the phase change of 5 °C CPCM was abrupt at 5 °C or so. Most of the 5 °C CPCM transformed from a solid into a liquid, and the 5 °C CPCM stored energy through absorption during the process. The temperature plateau was caused by the phase transition. Though the PCM of 5 °C CPCM absorbed heat continuously, the temperature changed slightly so that the 5 °C CPCM temperature rose slowly due to the influence of PCM. Temperature increased from 12 °C to 48 °C at the end of the heat exchange in the third section. From the DSC curves, the phase change peak temperature was about 12 °C, showing that the phase change of most PCM had finished.

In other words, during the temperature rise, 5 °C CPCM absorbed heat in a sensible form in the first and third sections, while in the second section, the absorbed heat form was latent heat and sensible.

#### 4.1.2. Specific Heat Capacity of 5 °C CPCM Specimen

**Figure 7:**
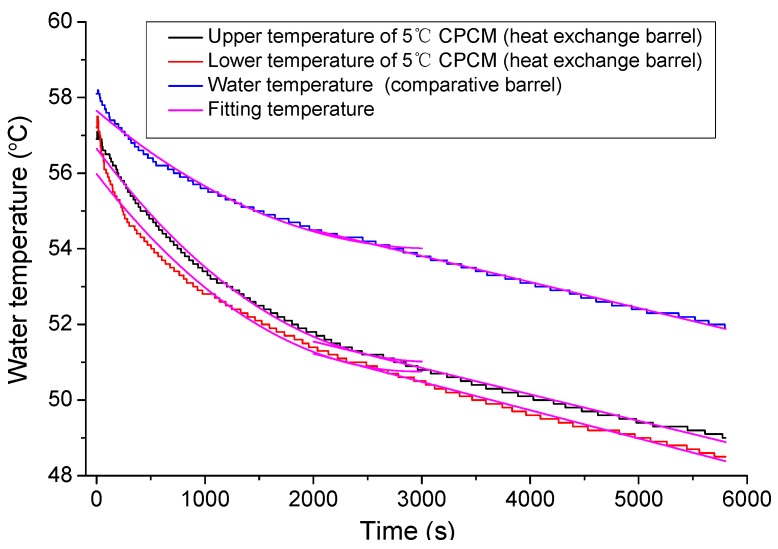
Initial and fitting temperature curves.

Based on temperature change curves of 5 °C CPCM, the temperature ranged from −18 °C to 44 °C and was divided by the 1 °C interval temperature to calculate the specific heat capacity. In Figure 7, firstly, the curve was concave. After 35 min, the curve linearly decreased, approximately; the reason may be that the decreasing rate of water temperature reduced continuously. Finally, the curve became stable. One reason may be that the temperature difference was larger in the initial period than the other periods, and heat exchange was more intense.

After trial fitting, the fitting function of temperature during 0–3000 s used an exponential function, $T=e^{a+bt+ct^{2}}$ while the fitting function of temperature from 2000 s to final used a linear function, $T=kt+T_{0}$; *t* represents time. The initial and fitting temperature curves are shown in Figure 7.

After deriving the initial fitting function, this study calculated the point of 2000~3000 s with the same derivation. The two curves were fine-tuned until the two function values of the points were equal. A continuous water temperature change curve was obtained by this break point.

Because the fitting curve error was large during the initial 3–4 min, the calculation of the specific heat capacity began with the 200th second. As shown in Figure 7, the upper and lower temperature of 5 °C CPCM gradients were consistent so that the average of the upper and lower water temperatures was selected as the water temperature of the heat exchange barrel. 

**Figure 8:**
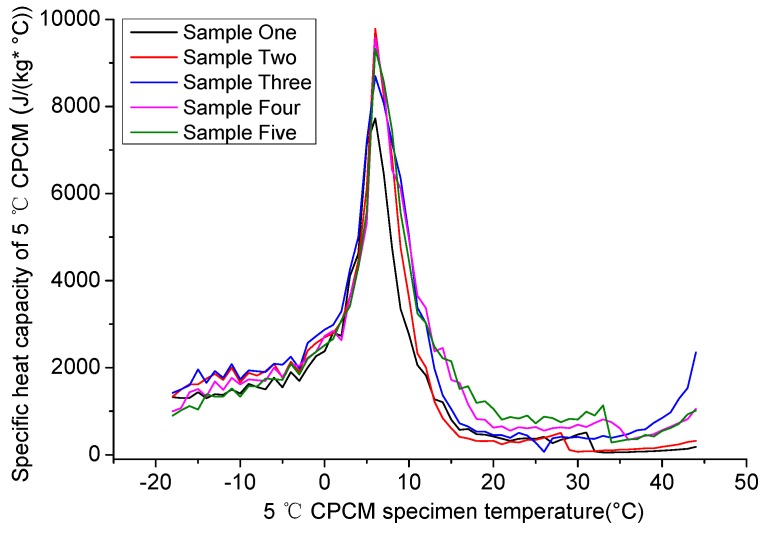
The curve of 5 °C CPCM specimen specific heat capacity changing along with temperature.

The fitting temperature data and Equation (3) were used to calculate the specific heat capacity of the 5 °C CPCM specimen. The curves of the change in specific heat capacity with temperature are shown in Figure 8.

In Figure 8, the curve was divided into three sections. The boundary points were the maximum of the curve increase and the minimum of the curve decrease.

During the first section, the initial rising rate was small, and, along with the phase transition, the rising rate increased significantly. Beginning at about −5 °C, the specific heat capacity increased sharply, and reached the peak at 7 °C with the maximum specific heat capacity focusing on 8000~10000 J/(kg·°C). At that point, PCM transformed from solid to liquid acutely. During this period, the 5 °C CPCM was solid the entire time, and the specific heat capacity increased with the rise in temperature, which meets the general law of solid specific heat capacity changing with temperature.

During the second section, the specific heat capacity decreased quickly. Approaching 25 °C, the specific heat capacity reached the minimum, which was lower than before the phase change. The specific heat capacity decreased along with the decreasing rate of heat absorption.

The initial temperature of the third section was almost the same as the temperature change in the final phase change. During this section, PCM was completely transformed into a liquid. Since liquid has a low density, a larger degree of freedom and lower entropy, the specific heat capacity of liquid is lower than that of solid for the same material. The curve changing behaviors were similar to the above rule, and the specific heat capacity of this section increased along with the rise in temperature.

Based on the curves of the change in specific heat capacity, the specific heat capacity first increased and reached the maximum at the initial phase change temperature with an increase in temperature. Then it decreased and reached the minimum at the temperature of the final phase change. Finally, the specific heat capacity slowly increased when compared with the first section and the second section.

#### 4.1.3. Recommended Parameters of 5 °C CPCM Specimen Specific Heat Capacity

From the changing curves of the specific heat capacity along with the rise in temperature, the specific heat capacity was different on a large temperature interval. Therefore, the *c*(*T*) function was recommended as the parameters for specific heat capacity via the regression equation.

Three regression equations were applied to fit the three sections in Figure 8. Temperature intervals of the regression equation were −18~7 °C, 7~25 °C and 25~44 °C, respectively. This study used the exponential function $c=y_{0}+Ae^{R_{0}T}$ as the regression equation. *y*_0_, A, and *R*_0_ were constant. According to the regression results, *R*^2^ of 25~44 °C was close to 0.8; the *R*^2^ of the other was larger than 0.9, proving that the regression results can reflect the curve accurately. *F* was larger than *P*, reflecting the reliability of the regression results. Apparently, the regression equations were significant, stable and credible, reflecting the actual specific heat capacity.

The recommended specific heat capacity parameters are described in Equation (4).

(4) $$c=\{\begin{array}{ll} 1439.942+1043.397e^{0.291T} & \left(-18\sim 7\;{}^{\circ}\text{C}\right)\\ 367.281+80135.913e^{-0.313T} & \left(7\sim 25\;{}^{\circ}\text{C}\right)\\ 387.166+1.147\times 10^{-8}e^{0.563T} & \left(25\sim 44\;{}^{\circ}\text{C}\right) \end{array}$$

### 4.2. Results and Analysis of Asphalt Mixture Mixed with 5 °C CPCM

#### 4.2.1. Temperature Changing Behaviors of Asphalt Mixture Mixed with 5 °C CPCM

**Figure 9:**
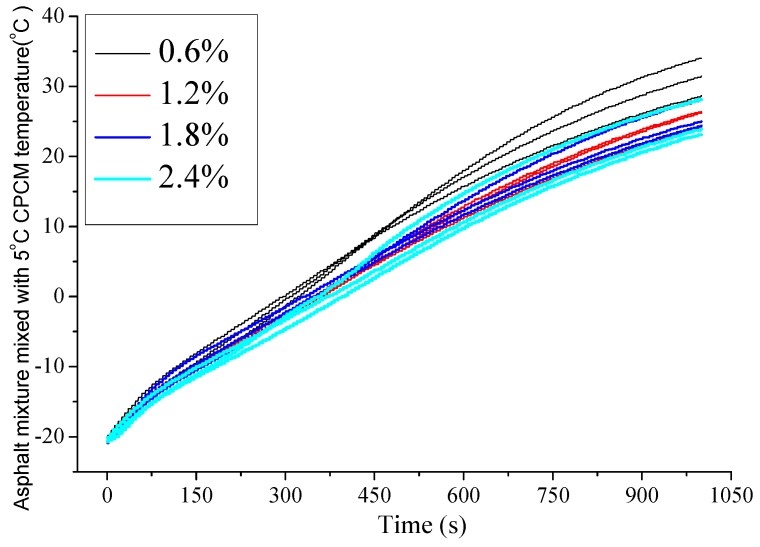
Temperature curves of asphalt mixture mixed with 5 °C CPCM.

The temperature change curves of the asphalt mixture mixed with 5 °C CPCM with the four different mass concentrations are shown in Figure 9.

As shown in Figure 9, in the middle of all the curves, there existed a slight concave, stating that the change in the heating rate was nonlinear; this was similar to the heat-rising curves of the 5 °C CPCM. The concave is slight because compared with the Marshall specimen mass, the 5 °C CPCM mass is too small to significantly affect asphalt mixture, which leads to a more subtle temperature plateau. The changing rules of all the specimens were almost the same. Because water temperature decreased by the heat storage of 5 °C CPCM, the higher the dosage of 5 °C CPCM, the lower the temperature curve, and the lower the final heat exchange temperature. Due to the low dosage of 5 °C CPCM, there was little influence of 5 °C CPCM on the specific heat capacity of the asphalt mixture, and the delay in temperature rise was insignificant.

#### 4.2.2. Specific Heat Capacity of Asphalt Mixture Mixed with 5 °C CPCM

**Figure 10:**
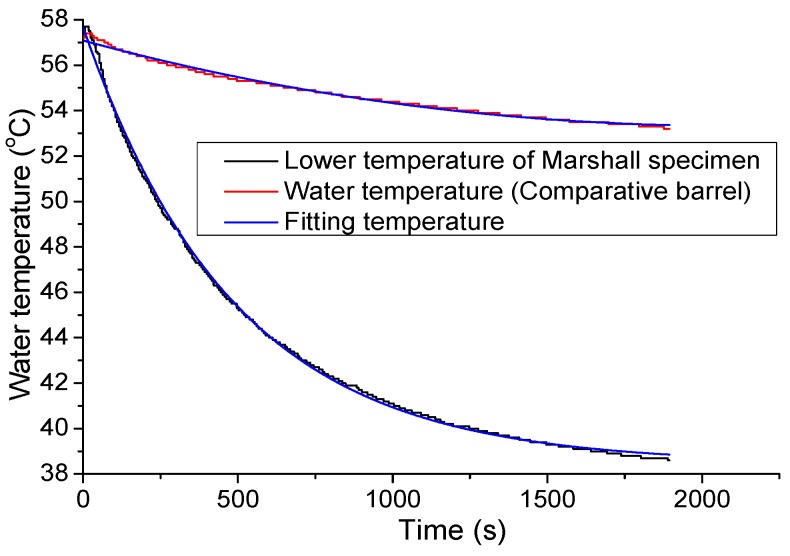
Temperature and fitting temperature curves of 2#.

The volume of the Marshall specimen is larger than that of the 5 °C CPCM specimen, which leads to a difference in the upper temperature and lower temperature of the specimen. Since the upper water spilled over the specimen a little, the Marshall specimen selected a lower temperature as the heat exchange temperature. 2# was taken as an example to correct the lower temperature. Compared to the 5 °C CPCM specimen, the heat exchange process was shorter, thus the fitting function of the comparative water temperature was $T=e^{a+bt+ct^{2}}$, and the fitting function of the lower temperature of the Marshall specimen was $T=y_{0}+Ae^{tR_{0}}$. The initial temperature and fitting temperature curves are shown in Figure 10.

The calculation time of the specific heat capacity of the Marshall specimen was similar to 5 °C CPCM, which began from the 200th second.

**Figure 11:**
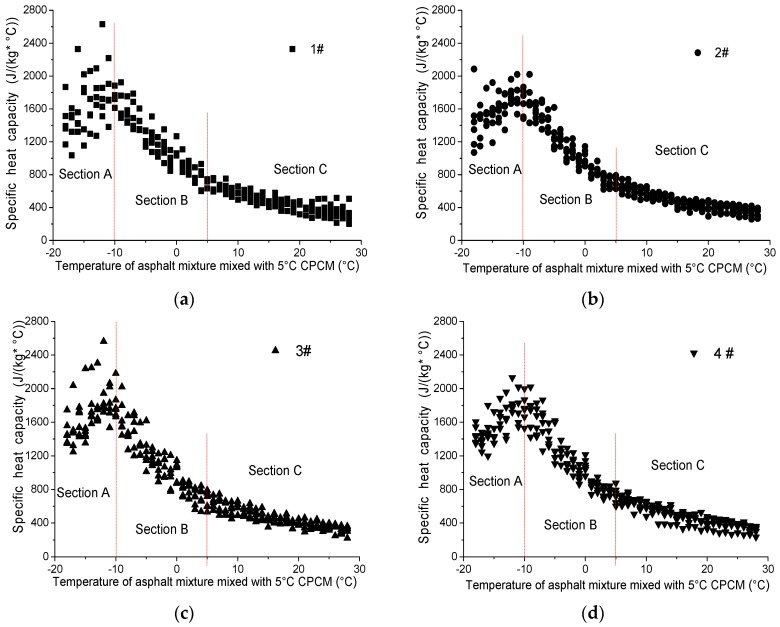
Scatter diagrams of specific heat capacity of asphalt mixture changing with temperature: (**a**) 0.6% dosage of 5 °C CPCM; (**b**) 1.2% dosage of 5 °C CPCM; (**c**) 1.8% dosage of 5 °C CPCM; and (**d**) 2.4% dosage of 5 °C CPCM.

The scatter diagrams of the specific heat capacity of the asphalt mixture changing with the temperature of four different mass concentrations are shown in Figure 11; the temperature ranged from –18 to 28 °C. The specific heat capacity of the asphalt mixture was calculated by the fitting temperature data and Equation (3).

According to Figure 11, each curve was divided into three sections; the temperature of Section A ranged from −18 to −10 °C, the temperature of Section B ranged from −10 to 5 °C and Section C ranged from 5 to 28 °C.

As shown in Figure 11, points in Section A were relatively scattered, as 5 °C CPCM had not started the phase transition process. As for Section B, with the increase in temperature, the specific heat capacity decreased linearly, approximately, in which 5 °C CPCM started to phase change slowly but had not reached the stage of quick heating. During Section C, the specific heat capacity decreased continuously; the slope was obviously lower than that of Section B. At that point, the temperature of the 5 °C CPCM phase change process ranged from 5 to 25 °C, and the key temperature range of the phase change process was 5 to 12 °C.

#### 4.2.3. Recommended Parameters of Asphalt Mixture Mixed with 5 °C CPCM

Linear regression was used to analyze the three sections, and the calculation method was the least squared method.

As with the increasing temperature, the data in Section A were scattered. Therefore, this study used the linear approximation method to fit the raw data in Section A. The initial linear regression results showed that the slopes of all the curves were larger than 0, which proves that the specific heat capacity increased with rising temperature. However, the corresponding determinate coefficients were very small, showing that the fitting degree of the regression function was unsatisfactory and the fitting function inaccurately reflected the real changes in temperature. To eliminate this adverse effect, the data were filtered using the statistical analysis method. Then, the representative data were linearly regressed. The regression results are shown in Table 2.

As illustrated in Table 2, the regression improved significantly, which reflects the actual changing rule of the specific heat capacity.

As for Section B, the specific heat capacity of temperatures from −10 to 5 °C was regressed linearly. The linear regression function is shown in Table 3.

From Table 3, it can be seen that *R*^2^ is larger than 0.8, which proves that the fitting degree is satisfied; meanwhile, *F* was larger than *P*, and *P* was 0, showing that the regression function was highly significant.

As for Section C, the regressed specific heat capacity of the mixture of the four asphalt groups ranged from 5 to 28 °C, linearly. The regression function is shown in Table 4.

The regression function of Section C was similar to Section B, showing that the regression function was significant.

Above all, because the low dosage of 5 °C CPCM has little influence on the specific heat capacity of the asphalt mixture, the average regression function coefficients were selected as a recommendation for the specific heat capacity of the asphalt mixture. The recommendations of the specific heat capacity of the asphalt mixture mixed with 5 °C CPCM are shown in Table 5.

## 5. Conclusions

In this paper, the heat exchange system and the data acquisition system were used to test the temperature change behaviors and the specific heat capacities of 5 °C CPCM and asphalt mixture mixed with 5 °C CPCM.

It is important to note that the 5 °C CPCM temperature-rise curve has an obvious temperature plateau while that of the asphalt mixture mixed with 5 °C CPCM does not have a temperature plateau. The temperatures of boundary points on the temperature-rise curve are due to the phase change temperature and the thermal compensation on the DSC scanning curve. As for the 5 °C CPCM, with an increase in temperature, the specific heat capacity first increased and reached the maximum at the initial phase change temperature, then decreased and reached the minimum at the final phase change temperature. Finally, the specific heat capacity increased slowly. With increasing temperature, the specific heat capacity of the asphalt mixture first linearly increased and then decreased linearly. It can be concluded that the low dosage of 5 °C CPCM has little influence on the specific heat capacity of the asphalt mixture, and the delay in the rise in temperature is insignificant. This may be due to different masses. When compared with the mass of the Marshall specimen, the 5 °C CPCM mass is too small to significantly affect asphalt mixture, which leads to the temperature plateau being less obvious. This is of some applicable significance in studying the thermoregulation mechanism and efficiency of CPCM on asphalt mixture. Meanwhile, this work provides parameters and a theoretical foundation for further studies. The limitation of this study is that the specific heat capacity was calculated by multiple fittings of the original data so that there exists cumulative error. Therefore, the processing of original data needs further improvement.

## Figures and Tables

**Table 1:** Water *C*p values of different temperature.

Temperature	Water *C*p Values
0 °C	4212 J/(kg·°C)
20 °C–60 °C	4179 J/(kg·°C)
100 °C	4220 J/(kg·°C)

**Table 2:** Regression function of Section A.

*c* = *b* + *kT*	*y* Intercept		Slope		*R*^2^	*F*	*P*
	*b*	σ	*k*	σ			
0.6%	2266.25306	98.88348	45.34586	6.98557	0.64140	42.13775	1.5718E-6
1.2%	2192.90216	71.85695	42.42836	4.90949	0.73918	74.68605	5.58995E-9
1.8%	2000.60122	78.97311	47.90939	5.48087	0.71288	72.43339	5.14278E-9
2.4%	2250.58206	56.12908	46.13190	4.36640	0.81838	80.91606	1.0 0332E-8

**Table 3:** Regression function of Section B.

*c* = *b* + *kT*	*y* Intercept		Slope		*R^2^*	*F*	*P*
	*b*	σ	*k*	σ			
0.6%	1007.76487	14.55219	−67.51472	2.75011	0.8775	602.69612	0
1.2%	982.59219	12.80987	−69.688	2.42084	0.89124	828.67598	0
1.8%	889.48133	21.90015	−72.15694	4.13874	0.85834	603.96246	0
2.4%	995.88062	22.68982	−72.37224	4.28797	0.85024	584.86557	0

**Table 4:** Regression function of Section C.

*c* = *b* + *kT*	*y* Intercept		Slope		*R^2^*	*F*	*P*
	*b*	σ	*k*	σ			
0.6%	742.48076	12.11364	−16.21584	0.68991	0.8375	552.45665	0
1.2%	698.87274	10.66406	−13.62437	0.58443	0.80191	543.46803	0
1.8%	617.33916	10.80598	−11.88699	0.60392	0.84478	487.42904	0
2.4%	751.29099	17.39458	−15.88174	0.93331	0.83264	389.56278	0

**Table 5:** Regression function of the asphalt mixture mixed with 5 °C CPCM.

*c* = *b* + *kT*	*y* Intercept	Slope
Section A	2177.585	45.454
Section B	968.930	−70.433
Section C	702.496	V14.402
